# Hair Sheep Blood, Citrated or Defibrinated, Fulfills All Requirements of Blood Agar for Diagnostic Microbiology Laboratory Tests

**DOI:** 10.1371/journal.pone.0006141

**Published:** 2009-07-03

**Authors:** Ellen Yeh, Benjamin A. Pinsky, Niaz Banaei, Ellen Jo Baron

**Affiliations:** 1 Department of Pathology, Stanford University School of Medicine, Stanford, California, United States of America; 2 Clinical Microbiology Laboratory, Stanford Hospital and Clinics, Palo Alto, California, United States of America; Columbia University, United States of America

## Abstract

**Background:**

Blood agar is used for the identification and antibiotic susceptibility testing of many bacterial pathogens. In the developing world, microbiologists use human blood agar because of the high cost and inhospitable conditions for raising wool sheep or horses to supply blood. Many pathogens either fail to grow entirely or exhibit morphologies and hemolytic patterns on human blood agar that confound colony recognition. Furthermore, human blood can be hazardous to handle due to HIV and hepatitis [1], [2]. This study investigated whether blood from hair sheep, a hardy, low-maintenance variety of sheep adapted for hot climates, was suitable for routine clinical microbiology studies.

**Methods and Findings:**

Hair sheep blood obtained by jugular venipuncture was anticoagulated by either manual defibrination or collection in human blood bank bags containing citrate-phosphate-dextrose. Trypticase soy 5% blood agar was made from both forms of hair sheep blood and commercial defibrinated wool sheep blood. Growth characteristics, colony morphologies, and hemolytic patterns of selected human pathogens, including several streptococcal species, were evaluated. Specialized identification tests, including CAMP test, reverse CAMP test, and satellite colony formation with *Haemophilus influenzae* and *Abiotrophia defectiva* were also performed. Mueller-Hinton blood agar plates prepared from the three blood types were compared in antibiotic susceptibility tests by disk diffusion and E-test.

**Conclusions:**

The results of all studies showed that blood agar prepared from citrated hair sheep blood is suitable for microbiological tests used in routine identification and susceptibility profiling of human pathogens. The validation of citrated hair sheep blood eliminates the labor-intensive and equipment-requiring process of manual defibrination. Use of hair sheep blood, *in lieu* of human blood currently used by many developing world laboratories and as an alternative to cost-prohibitive commercial sheep blood, offers the opportunity to dramatically improve the safety and accuracy of laboratory diagnosis of pathogenic bacteria in resource-poor countries.

## Introduction

Agar supplemented with 5% blood is a general growth medium routinely used in the clinical microbiology laboratory for identification of pathogenic bacteria [3]. This enriched medium supports the growth of many pathogenic organisms but, at the same time, allows differential characterization of these bacteria based on their hemolytic patterns. For example, different species of streptococci can be classified according to each of 3 types of hemolytic reactions. β-hemolytic strains completely hemolyze the red cells, generating a clear zone around the colony. Pathogens which demonstrate β-hemolysis include *S. pyogenes* (Group A streptococcus), the organism responsible for strep throat and its many sequelae such as rheumatic fever and post-streptococcal glomerulonephritis, as well as *S. agalactiae* (Group B streptococcus), an important agent in neonatal sepsis. Partial hemolysis causing a green discoloration of the agar is seen in α-hemolytic strains, which include the causative organism of most community-acquired pneumonias and meningitis, *S. pneumoniae.* Strains such as *S. bovis*, which causes an infection associated with gastrointestinal cancers, do not hemolyze blood and are classified as γ-hemolytic. Determining the growth characteristics, colony morphologies, and hemolytic patterns on blood agar plates are an important initial step in the identification of pathogenic bacteria.

Currently, clinical laboratories in many resource-poor countries are unable to prepare blood media from animal blood, such as sheep and horse, due to the high cost and inhospitable climate for raising these animals [4]. Instead, developing world laboratories commonly utilize human blood for preparation of microbiological media, either expired blood from hospital blood banks or blood from volunteers, often the laboratory technologists themselves. Given the high prevalence of HIV, hepatitis, and other blood-borne infections in the developing world, the preparation of human blood media poses considerable infection risk to laboratory workers. Furthermore, many pathogenic bacteria exhibit altered growth and hemolytic patterns when grown on agar plates prepared from human blood compared to animal blood [1], [2], resulting in a serious potential for misdiagnosis of infectious diseases.

**Figure 1:**
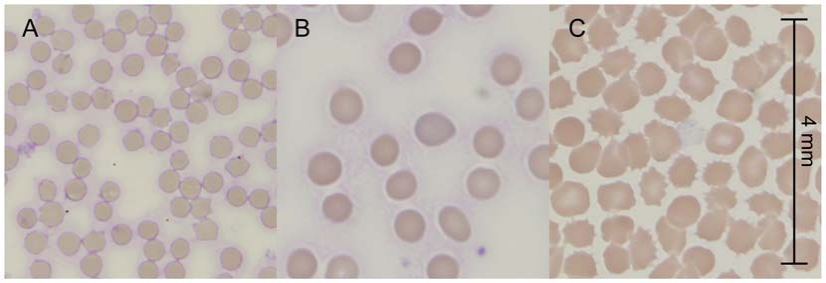
Comparison of red blood cells from sheep, human, and expired blood bank blood. (A and B) Sheep red blood cells (A) are smaller in size compared to human red cells (B). (C) Human red blood cells after >45 days of storage in the blood bank demonstrate significant formation of echinocytes, spherical spiculated cells, that are distinctly different from the biconcave disk of normal red cells. All panels are shown with the same scale as indicated in (C).

The altered growth and hemolytic reactions of bacteria grown on animal versus human blood media may be due to differences in the morphology and membrane composition of the red cells that affect their ability to be lysed by bacterial hemolysins. For example, we observed human red cells to be substantially larger than sheep red cells in fresh blood samples (Figure 1A and 1B). Discrepancies in the results of the CAMP test have also been attributed to differences in the sphingomyelin content of the membrane. The CAMP reaction, which is commonly used to identify Group B streptococcus, depends on two factors: CAMP factor secreted by Group B streptococcus and a sphingomyelinase secreted by *Staphylococcus aureus* [5]–[7]. Hydrolysis of sphingomyelin in the red cell membrane sensitizes the erythrocyte to the lytic action of CAMP factor, resulting in an area of enhanced hemolysis when Group B streptococcus is grown near *S. aureus*. Sheep red cells with sphingomyelin content as high as 51% yield the best results in the CAMP test while human cells, with only 26% sphingomyelin content, do not support the CAMP reaction at all [2], [8].

The use of expired blood from blood banks is even more problematic because red cells are known to undergo many well-characterized morphologic and biochemical changes during storage. These storage lesions include oxidative damage to membrane lipids and proteins, decreased pH, depletion of ATP, loss of 2,3-diphosphoglycerate (2,3-DPG), and increased extracellular potassium due to dysfunction of the Na^+^-K^+^ ATPase [9], [10]. The perturbation of the normal red cell structure and biochemical environment increases with longer duration of storage. At the end of its shelf life (typically 45 days), almost 80% of stored red cells have transformed into spherical spiculated cells, known as echinocytes, that are distinctly different from the normal biconcave structure of fresh red cells (Figure 1C). These echinocytes demonstrate a significant decrease in their membrane deformability. These morphologic and functional changes associated with expired blood are likely to cause further differences in the growth and hemolytic reactions of bacteria grown on expired human blood agar versus fresh animal blood agar.

To improve both laboratory safety and diagnostic accuracy, an alternative to the use of human blood for media preparation in developing world laboratories is desperately needed. Any potential solution would need to address the existing hurdles to the use of media prepared from animal blood, including access to suitable animals as a blood source, the cost-effectiveness of raising such animals, and the ease of preparation of the blood. In particular, although wool sheep and horse blood are the standard for blood agar prepared in North America and Europe, respectively, wool sheep do not thrive in the warm, dry climates of many developing world countries while horse husbandry is extremely cost-prohibitive [3], [4]. Furthermore, the laborious process of defibrination by manual shaking of the blood during collection in order to prevent clot formation is a serious deterrent to potential suppliers. Recently, Russell *et al* demonstrated that citrate could be used as an anticoagulant *in lieu* of defibrination for preparation of sheep blood for agar plates. The citrated sheep blood, which was simply collected in blood bags containing citrate, was equivalent to defibrinated sheep blood and superior to citrated human blood in growth studies and disk diffusion antibiotic susceptibility tests of *S. pneumoniae*, Group A streptococcus, and *S. aureus* [1]. However, it is not known how citrated sheep blood would perform compared to defibrinated blood in more specialized microbiology tests used for bacterial identification such as satellite formation and CAMP tests or in penicillin susceptibility by E-test for *S. pneumoniae*, a critical assay for which no other method is currently practical.

In the current study, we investigated the use of hair sheep blood agar for bacterial identification and susceptibility testing. Hair sheep are a practical alternative source of animal blood for agar preparation since they are related to but distinct from wool sheep, adapted for the warmer climates of many developing world countries, and more cost-effective to maintain [11], [12]. Both defibrinated and citrated hair sheep blood were compared with standard blood media prepared from defibrinated wool sheep blood in growth studies and specialized microbiology tests. Accuracy of antibiotic susceptibility testing was also evaluated with drug-resistant clinical strains. Our results demonstrate that blood agar prepared from citrated hair sheep blood yielded reliable results for routine identification and susceptibility profiling of non-fastidious human pathogens. We propose that the use of citrated hair sheep blood offers a safe, cost-effective, and diagnostically accurate alternative for clinical microbiological testing that has the potential to dramatically improve infectious disease diagnosis in the developing world.

## Methods

### Ethics Statement: Maintenance and handling of sheep

All animal handling was conducted according to the approved Institutional Animal Care & Use Committee guidelines, in concordance with the Guide for the Care and Use of Agricultural Animals in Agricultural Research and Teaching, which is available to download at http://www.fass.org/page.asp?pageID=216 and the Guide to the Care and Use of Laboratory Animals, available to download at http://www.nap.edu/openbook.php?record_id=5140&page=82. The sheep were raised in open pasture with freely available water (changed daily) and protein feed supplement to support good nutrition, which is especially important if the animals are to be maintained and bled periodically. The sheep are handled daily, checked annually for infectious diseases, and otherwise maintained in a humane manner but consistent with sound farm-production practices. Sheep maintained for bleeding can also be used to produce milk for cheese, and ultimately as a source of meat. These sheep were scheduled for slaughter immediately after the experiment was completed. Sheep should be handled gently but firmly and they should be constrained during the bleeding process so that they feel secure and to prevent sudden movements. In most cases, the sheep will not even be aware when the needle enters the vein.

### Media and reagents

Trypticase-soy and Mueller-Hinton agar media were purchased from BD Microbiology Products (Sparks, MD). Antibiotic discs and E-test strips were obtained from BD and AB Biodisk,/bio Merieux (Solna, Sweden), respectively.

### Blood collection and agar plate preparation

Hair sheep blood was obtained by venipuncture via the jugular vein (yielding 350 mL of blood per animal). An area approximately 100 cm^2^ on the front of the animal's chest was prepared by generously spraying with 70% ethanol. The hair was not clipped or shaved, although clipping is recommended to allow better visualization of the vein and to prevent contamination. Shaving is neither necessary nor recommended. The blood was anticoagulated by either manual defibrination or by addition of citrate. For defibrination, blood was collected in a sterile bottle containing 9 cc of 4 mm glass beads with constant manual agitation during the bleeding process. For citrated blood, blood was collected in a standard human blood bank collection bag containing citrate-phosphate-dextrose at a ratio of 1.4 mL of citrated anticoagulant (14 mM citrate, 14 mM phosphate, 129 mM dextrose) per 10 mL of blood. To obtain the correct ratio of anticoagulant to blood, 21 mL of the citrate suspension was aseptically removed from a standard 500 mL human blood collection bag prior to use to yield the appropriate volume of anticoagulant for the expected 350 ml of blood. The blood bag was weighed periodically during the bleeding on a metric manual kitchen scale to verify the volume of blood.

Trypticase-soy and Mueller-Hinton agar media were prepared and supplemented with 5% blood either citrated hair sheep blood, defibrinated hair sheep blood, or defibrinated wool sheep blood (obtained from HemoStat Laboratories, Dixon, CA). Freshly poured plates were incubated overnight at 37°C. Those that showed contamination were discarded (3 out of a total of 85 plates prepared from hair sheep blood).

### Bacterial strains

Bacterial strains used in this study were obtained either from clinical specimens or purchased from American Type Culture Collection (ATCC) as indicated in Table 1. Fresh subcultures of each were diluted to 0.5 McFarland turbidity in sterile water and used for inoculation of agar plates.

**Table 1:** Sources of bacterial strains.

Organism	Source
*Abiotrophia defectiva*	Clinical strain
*Arcanobacterium haemolyticum*	Clinical strain
*Haemophilus influenzae*	ATCC 49247
*Listeria monocytogenes*	Clinical strain
*Neisseria meningitidis*	Clinical strain
*Pasteurella multocida*	Clinical strain
*Staphylococcus aureus*	ATCC 29213 (for CAMP and satellite tests) and clinical strain (for D-test)
*Streptococcus agalactiae* (Group B)	Clinical strain
*Streptococcus anginosus*	Clinical strain
*Streptococcus pneumoniae*	ATCC 49619 and clinical strain
*Streptococcus pyogenes* (Group A)	Clinical strain

### Growth studies, CAMP, and satellite tests

Bacteria were plated in duplicate using a 10 µL loop taken from identical suspensions onto trypticase-soy agar plates supplemented with each of the 3 different bloods (citrated hair sheep, defibrinated hair sheep, and commercial defibrinated wool sheep). For growth studies, the bacteria were streaked over the entire plate. For the CAMP and reverse CAMP test, a line of *S. aureus* ATCC 29213 was streaked in the middle of the agar plate. A line of Group B *streptococcus* (for the CAMP test) or *A. hemolyticum* (for the reverse CAMP test) was then placed perpendicular to the line of *S. aureus* but without allowing the lines to intersect. For the satellite test, *H. influenza* and *A. defectiva* were streaked all over the plate as in the growth studies. In addition, a linear streak of *S. aureus* ATCC 29213 was also placed in the middle of the inoculated plate.

### Kirby-Bauer disk diffusion and E-test

Mueller-Hinton agar plates supplemented with sheep blood were inoculated over the entire surface of the agar with drug-resistant bacteria by spreading with sterile cotton swabs. Antibiotic disks or E-test strips were placed directly on the inoculated plates. Zones of inhibition for the Kirby-Bauer disk diffusion were measured from the center of the disk to the edge of bacterial growth. For the double disk diffusion (D-test), a clindamycin disk was placed 2 cm apart from the erythromycin disk. For the E-test, the E-strip containing a gradient of antibiotic concentration was placed on an inoculated agar plate. Bacterial growth yielded an elliptical zone of inhibition ending at a point along the strip corresponding to the MIC value. All tests were performed in duplicate

## Results

### Comparison of hair sheep with commercial blood agar in growth studies

Several human pathogens were evaluated for their growth, colony morphology, and hemolytic reactions on hair sheep blood agar. On standard wool sheep blood media, both Group B streptococcus and Group A streptococcus grow as translucent colonies exhibiting β-hemolysis. Group A streptococcus colonies tend to be smaller (∼0.5 mm) with a sharply demarcated zone of hemolysis surrounding each colony and extending beyond the colony, while Group B streptococcus colonies are slightly larger (>0.5 mm) with soft β-hemolysis directly underneath the colony. These characteristics of their growth and hemolytic pattern on wool sheep blood agar were also apparent on both citrated and defibrinated hair sheep blood agar. Similarly, *Streptococcus anginosus* which is distinguished by its pinpoint colonies and characteristic sweet smell on standard blood agar could also be accurately identified by these features on hair sheep blood agar. The growth of two different strains of *S. pneumoniae*, ATCC 49619 and a clinical isolate, were also compared on standard media and hair sheep blood media. Both grew as transparent colonies and exhibited α-hemolysis typical of *S. pneumoniae* on citrated and defibrinated hair sheep agar. In addition, the clinical strain had a type 3 polysaccharide capsule mucoid phenotype on standard blood media that was also visible on hair sheep media. Finally, *Listeria monocytogenes* grew as large (1 mm) colonies with β-hemolysis limited to the area underneath the colony on both standard wool sheep blood agar and hair sheep agar.

### Accuracy of hair sheep blood agar in CAMP, reverse CAMP, and satellite colony formation tests

**Figure 2:**
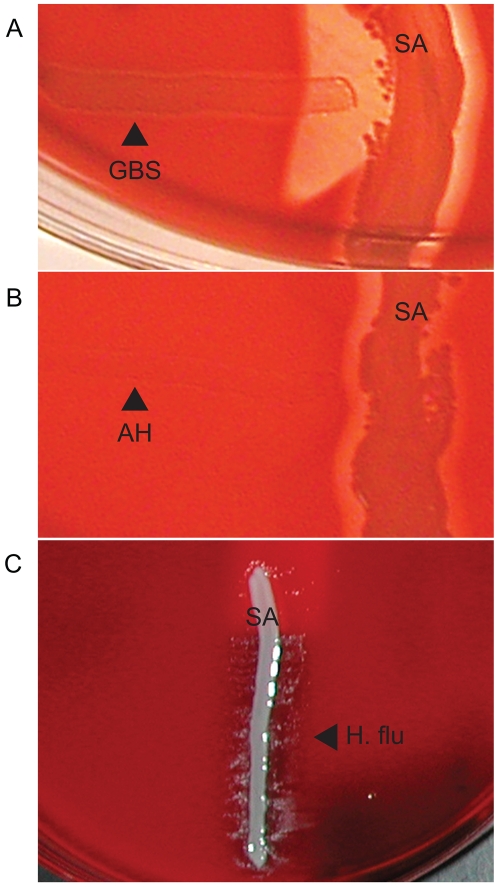
Identification of bacteria by specialized microbiological tests. (A) The CAMP test for identification of Group B streptococcus (horizontal streak, GBS) demonstrates an arrowhead shaped area of enhanced hemolysis when grown near *S. aureus* (vertical streak, SA). (B) The reverse CAMP test for identification of *Arcanobacterium haemolyticum* demonstrates an indentation in the linear area of hemolysis by *S. aureus* (vertical streak, SA) when it grows adjacent to *A. haemolyticum* (horizontal streak, AH). (C) The satellite test for identification of *Haemophilus influenzae* demonstrates growth of the organisms as small satellite colonies (arrowhead, H. flu) adjacent to *S. aureus* (vertical streak, SA). Similar satellite colony formation around *S. aureus* was seen for *Abiotrophia defectiva*. Similar results were seen on both defibrinated and citrated hair sheep blood agar; results are shown on citrated hair sheep blood agar plates.

In the clinical laboratory, certain organisms can be identified by specialized tests that take advantage of metabolic requirements or virulence factors characteristic of that pathogen. These tests are simple and low-cost to perform but can be invaluable in identification of certain pathogens. As discussed previously, Group B streptococci secrete CAMP factor that results in increased hemolysis when grown near *S. aureus* on blood agar [5]–[7]. As shown in Figure 2A, both defibrinated and citrated hair sheep blood agar demonstrated a positive CAMP reaction for Group B streptococcus, as seen by an arrowhead area of clearing on the agar (indicating enhanced hemolysis) where Group B streptococcus and *S. aureus* grow closest to each other. In contrast, *Arcanobacterium haemolyticum* secretes an inhibitory factor that suppresses the secondary β-hemolysin of *S. aureus* [13]. In the reverse CAMP test, there is an area of suppressed hemolysis by *S. aureus* when the colonies are growing adjacent to *A. haemolyticum*, seen as an indentation in the linear area of hemolytic clearing by *S. aureus* (Figure 2B).

Both *Haemophilus influenzae* and *Abiotrophia defectiva*, a species of nutritionally-deficient *Streptococcus*, are unable to grow on blood agar because they lack some factor required for survival on blood media. In the case of *H. influenzae* the missing factor is nicotinamide adenine dinucleotide (NAD), and for *A. defectiva* a source of pyridoxal must be provided [14]–[17]. *S. aureus* secretes both NAD and pyrdixoxal and therefore permits the growth of both organisms on blood agar as satellite colonies. In the satellite test, both *H. influenzae* and *A. defectiva* grew on hair sheep blood agar as small satellite colonies adjacent to *S. aureus* but nowhere else on the plate (Figure 2C).

### Determination of antibiotic susceptibility by disk diffusion, E-test, and the D-test for inducible clindamycin resistance

Assessment of antibiotic susceptibility of pathogens from clinical specimens is a critical function of the clinical microbiology laboratory since this testing guides the clinician's decisions about antimicrobial therapy. The disk diffusion method requires the least resources and delivers reliable results. Interpretive criteria for zones of inhibition for each organism/antimicrobial agent combination have been established (18). We tested strains of Group A streptococcus, Group B streptococcus, *Neisseria meningitidis*, and *Pasteurella multocida* by disk diffusion on hair sheep and wool sheep blood agar plates. As shown in Table 2, the measured zones of inhibition for different antibiotics for each of these organisms were similar on both media. Hair sheep Mueller-Hinton blood agar accurately determined the sensitive or resistant phenotype of these strains.

**Table 2:** Zones of inhibition (mm) as measured by disk diffusion and interpretation of resistance phenotype^1^^,^ 2 ^,^ 3.

		P	Cro	CC	E	CIP	OX	M	D	LVX	SXT
*Streptococcus pyogenes*	DS	19,20	19,19	14,14	16,16						
		NA	NA	R	I						
	DH	19,20	19,20	13,13	15,15						
		NA	NA	R	R						
	CH	19,20	20,20	13,14	15,15						
		NA	NA	R	R						
*Streptococcus agalactiae*	DS	16,16	16,16	11,12	12,12						
		NA	NA	R	R						
	DH	16,18	16,16	11,11	12,12						
		NA	NA	R	R						
	CH	16,16	16,17	11,11	12,12						
		NA	NA	R	R						
*Neisseria meningitidis*	DS	17,17	20,20			18,18		20,20			
		NA	NA			R		NA			
	DH	17,17	20,21			19,19		20,21			
		NA	NA			R		NA			
	CH	17,18	19,19			19,20		19,19			
		NA	NA			R		NA			
*Pasteurella multocida*	DS	14,14	17,19		10,12				11,14	19,20	19,19
		NA	NA		R				NA	NA	NA
	DH	15,14	17,19		10,10				11,11	14,15	15,15
		NA	NA		R				NA	NA	NA
	CH	14,14	17,18		11,12				13,16	15,18	15,18
		NA	NA		R				NA	NA	NA
*S. pneumoniae* ATCC 49619	DS			11,11	14,14	10,11	4,4				
				R	R	NA	I				
	DH			11,11	14,14	10,10	4,5				
				R	R	NA	I				
	CH			10,11	14,14	10,11	4,5				
				R	R	NA	I				
*S. pneumoniae* (clinical strain)	DS			12,12	15,15	8,8	12,12				
				R	R	NA	I				
	DH			11,12	14,15	8,8	11,12				
				R	R	NA	I				
	CH			11,11	13,14	8,8	12,12				
				R	R	NA	I				

Tests were performed in duplicate, and both measurements are reported for each organism/antibiotic.

1 DS, commercial defibrinated wool sheep blood; DH, defibrinated hair sheep blood; CH, citrated hair sheep blood.

2 P, penicillin G 10 U; Cro, ceftriaxone 30 µg; CC, clindamycin 2 µg; E, erythromycin 15 µg; CIP, ciprofloxacin 5 µg; M, meropenem 10 µg; D, doxycycline 30 µg; LVX, levofloxacin 5 µg; SXT, sulfamethoxazole-trimethoprim 1.25 µg/23.75 µg.

3 S, sensitive; R, resistant; I, indeterminate; NA, no interpretation available.

**Figure 3:**
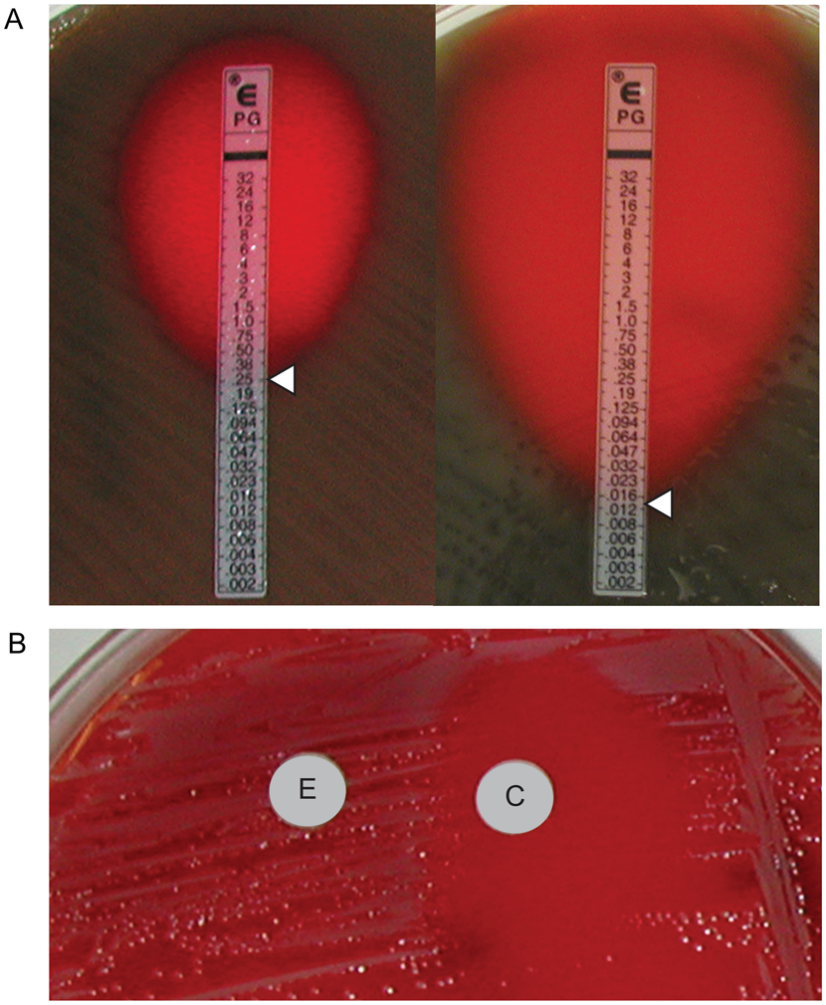
Use of hair sheep blood agar in antibiotic susceptibility testing. (A) Determination of MIC values for penicillin by E-test for a resistant (left) and a susceptible (right) strain of *S. pneumoniae*. (B) The D-test demonstrates inducible clindamycin resistance in a *S. aureus* strain carrying an inducible *erm* gene. A “D”-shaped area of clearing is seen due to increased growth around the clindamycin disk (labeled “C”) where it lies closest to the erythromycin disk (labeled “E”). Similar results were seen on both defibrinated and citrated hair sheep blood agar; results are shown on citrated hair sheep blood agar plates.

For *S. pneumoniae*, a zone of inhibition >2 cm surrounding a 1 µg oxacillin disk predicts penicillin sensitivity [18]. For zones <2 cm, the resistance phenotype cannot be determined by the disk diffusion method. We assessed the penicillin sensitivity of two strains of *S. pneumoniae* by disk diffusion: a penicillin-resistant/intermediate ATCC 49619 strain and a penicillin-sensitive clinical strain (Table 2). However, both were found to be <2 cm by Kirby-Bauer method. In these cases, penicillin susceptibility must be determined by measuring the minimal inhibitory concentration (MIC) using the E-test method. We were able to accurately determine the MIC values for both the penicillin-resistant/intermediate (0.25 µg/mL) and penicillin-sensitive (0.016 µg/mL) strain on hair sheep Mueller-Hinton blood agar which were the same as those measured on standard blood agar (Figure 3A).

Finally, we performed double disk diffusion, or D-test, on an erythromycin-resistant strain of *S. aureus* that also harbored inducible clindamyin resistance. These strains carry the *erm* gene which confers clindamycin resistance but whose expression is only induced in the presence of erythromycin [19], [20]. In the absence of erythromycin, the strain will appear to be clindamycin-susceptible, potentially leading to inadequate therapy if no further testing is performed. In the D-test, *S. aureus* is grown in the presence of both an erythromycin and clindamycin disk. Inducible expression of clindamycin resistance is observed by a blunting of the zone of inhibition formed around the clindamycin disk on the side closest to the erythromycin disk, forming a “D”-shaped clearing around the clindamycin disc. We were able to demonstrate a positive D-test for a resistant strain of *S. aureus* when grown on defibrinated and citrated hair sheep agar (Figure 3B).

## Discussion

Wool sheep blood and horse blood are the standards for blood media used in North America and Europe, respectively [3], [4]. However, both are impractical as sources of blood for microbiology media for many developing world laboratories, which use readily-available human blood instead despite concerns over its safety and diagnostic accuracy. Wool sheep are not adapted to the tropical climates typical of many of developing world countries and require docking of their tails and periodic shearing, a laborious procedure that yields no usable product for the owners in these regions [4], [11]. Horses are expensive to own and maintain and therefore not suitable for resource-poor areas. Other sources of animal blood have been explored for use in the clinical microbiology laboratory, however each presents its own challenges which preclude widespread usage. Goat blood is similar to sheep blood and yields results on blood agar that are diagnostically accurate [4], [21], [22]. However, goats require a large range area and are indiscriminate in their food consumption making them prone to acquiring infections. They are also less docile than sheep and thus difficult to handle. Pigs are easier to handle and maintain, however media prepared from pig blood does not yield acceptable results for some common microbiology tests [4].

In this study, we investigated the use of media prepared from hair sheep blood. Both wool and hair sheep are descended from an ancestral wild sheep, which had a coat of long hair fibers and a downy undercoat [11]. Sheep bred to enrich for the woolly undercoat, a valuable commodity in cold climates, became the dominant breeds worldwide and compose 90% of the world sheep population. However, there exists a group of breeds, known as hair sheep, which retain the more primitive characteristics [11], [12]. They thrive in warm, dry climates and have a higher tolerance for heat and humidity compared to wool sheep. 90% are found in Africa, and the rest in Latin America and the Caribbean. Because their wool sheds naturally, they do not need to be sheared, eliminating a costly and laborious step in their maintenance. Hair sheep are also hardier and more parasite resistant and do not require tail docking in order to prevent infection, another maintenance step required for keeping wool sheep. This resistance to infection is despite being kept at higher densities because hair sheep require less area for grazing. Their behavior is otherwise like that of wool sheep, and they are therefore very tractable animals. In the US, hair sheep are considered “easy care” sheep and are raised primarily as a source of meat [12].

Given their many favorable characteristics, hair sheep seem an ideal animal to serve as the source of animal blood to replace the use of human blood in clinical microbiology laboratories in the developing world. In our study, media prepared from both citrated and defibrinated hair sheep blood was shown to yield identical growth characteristics and hemolysis patterns for several pathogenic bacteria compared with standard blood agar prepared from wool sheep blood. In addition, hair sheep blood media was validated in several tests used for identification of pathogenic organisms as well as in antibiotic susceptibility testing essential in guiding therapy. Taken together, these results validate the diagnostic accuracy of hair sheep blood agar for use in routine identification and susceptibility profiling of common human pathogens. Furthermore, we extend the previous investigation by Russell *et al* using citrated sheep blood to demonstrate that citrated hair sheep blood performs well in more specialized tests, including the CAMP test and the critical E-test for penicillin sensitivity of *S. pneumoniae*, and can be substituted for defibrinated sheep blood which is more difficult to prepare [1]. Given the importance of accurate microbiological diagnosis and the imperative need for a better, safer, and cost-effective alternative to the use of human blood for resource-poor countries, we believe these findings have important clinical implications.

Hair sheep may already be available in some developing world countries, otherwise they could easily be imported given their adaptability to tropical environments. A 50–60 lb sheep may yield about 350 ml of blood every 6 weeks, which can be directly collected into standard veterinary blood bags that contain the proper amount of citrate for anticoagulation while preserving the sterility of the blood during collection. Since manual defibrination of the blood is not necessary, the process of blood collection is much less laborious and also does not require an autoclave at the collection facility to sterilize equipment for blood collection. The simplification introduced by use of citrated blood would allow small commercial operations or individual farmers to produce laboratory-grade blood to supply local laboratories with minimal upfront investment in expensive equipment or need for special training of workers.

This strategy for making available hair sheep blood agar is already being implemented in Botswana, where two regional laboratories will produce blood agar plates for all of the country's diagnostic microbiology laboratories. Before it was established that citrated blood would be acceptable, the country's veterinary health sciences department was unwilling to commit the labor and materials needed to prepare defibrinated blood, and laboratories continued to use expired human blood bank blood leading to lack of recovery or detection of many important pathogens and lower overall reliability of microbiology laboratory tests. It is now possible for Botswana to serve as a role model for the implementation of hair sheep blood agar in developing world clinical microbiology labs. If successfully implemented, blood agar prepared from hair sheep blood would have a large impact in improving healthcare delivery in the developing world.
